# Lower inhibitory control interacts with greater pain catastrophizing to predict greater pain intensity in women with migraine and overweight/obesity

**DOI:** 10.1186/s10194-017-0748-8

**Published:** 2017-03-29

**Authors:** Rachel Galioto, Kevin C. O’Leary, J. Graham Thomas, Kathryn Demos, Richard B. Lipton, John Gunstad, Jelena M. Pavlović, Julie Roth, Lucille Rathier, Dale S. Bond

**Affiliations:** 1 Department of Psychiatry and Human Behavior, Alpert Medical School of Brown University, Providence, RI USA; 2 Rhode Island Hospital, Providence, RI USA; 3 Neuropsychology Program, Rhode Island Hospital, 593 Eddy St., Providence, RI 02903 USA; 4 The Miriam Hospital Weight Control and Diabetes Research Center, Providence, RI USA; 5 Department of Neurology, Albert Einstein College of Medicine/Montefiore Medical Center, Bronx, NY USA; 6 Department of Psychological Sciences, Kent State University, Kent, OH USA; 7 Department of Neurology, Alpert Medical School of Brown University/Rhode Island Hospital, Providence, RI USA

**Keywords:** Migraine, Overweight, Obesity, Inhibitory control, Pain catastrophizing

## Abstract

**Background:**

Pain catastrophizing (PC) is associated with more severe and disabling migraine attacks. However, factors that moderate this relationship are unknown. Failure of inhibitory control (IC), or the ability to suppress automatic or inappropriate responses, may be one such factor given previous research showing a relationship between higher PC and lower IC in non-migraine samples, and research showing reduced IC in migraine. Therefore, we examined whether lower IC interacts with increased PC to predict greater migraine severity as measured by pain intensity, attack frequency, and duration.

**Methods:**

Women (n = 105) aged 18–50 years old (M = 38.0 ± 1.2) with overweight/obesity and migraine who were seeking behavioral treatment for weight loss and migraine reduction completed a 28-day smartphone-based headache diary assessing migraine headache severity. Participants then completed a modified computerized Stroop task as a measure of IC and self-report measures of PC (Pain Catastrophizing Scale [PCS]), anxiety, and depression. Linear regression was used to examine independent and joint associations of PC and IC with indices of migraine severity after controlling for age, body mass index (BMI) depression, and anxiety.

**Results:**

Participants on average had BMI of 35.1 ± 6.5 kg/m^2^and reported 5.3 ± 2.6 migraine attacks (8.3 ± 4.4 migraine days) over 28 days that produced moderate pain intensity (5.9 ± 1.4 out of 10) with duration of 20.0 ± 14.2 h. After adjusting for covariates, higher PCS total (β = .241, SE = .14, *p* = .03) and magnification subscale (β = .311, SE = .51, p < .01) scores were significant independent correlates of longer attack duration. IC interacted with total PCS (β = 1.106, SE = .001, *p* = .03) rumination (β = 1.098, SE = .001, *p* = .04), and helplessness (β = 1.026, SE = .001, *p* = .04) subscale scores to predict headache pain intensity, such that the association between PC and pain intensity became more positive at lower levels of IC.

**Conclusions:**

Results showed that lower IC interacted with higher PC, both overall and specific subcomponents, to predict higher pain intensity during migraine attacks. Future studies are needed to determine whether interventions to improve IC could lead to less painful migraine attacks via improvements in PC.

## Background

Migraine is a prevalent neurologic disease characterized by severe recurrent headache pain [1] and is associated with substantial functional and quality of life impairments [2]. Psychological factors, particularly those specific to pain, are known to contribute to greater migraine severity (i.e., pain intensity, attack frequency, and duration). Specifically, pain catastrophizing, which is defined as a negative mental set in response to pain involving difficulty inhibiting pain related thoughts (rumination), exaggerating the negative consequences of pain (magnification), and negatively evaluating one’s ability to cope with pain (helplessness) [3, 4], is associated with migraine attacks that are more frequent, severe, and longer in duration, and with greater headache-related disability and impairments in quality of life [5–7]. Additionally, individuals with clinically significant levels of pain catastrophizing are at greater risk for chronic migraine [5].

While the above findings demonstrate the importance of pain catastrophizing in the context of migraine, very little is known about factors that contribute to higher levels of pain catastrophizing in individuals with migraine. It is possible that lower inhibitory control, defined as ability to suppress automatic and/or inappropriate thoughts or behaviors, may influence the frequency or degree of pain catastrophizing in persons with migraine. Specifically, lower inhibitory control may make it more difficult for participants to control pain-related thoughts, resulting in thoughts that are more uncontrollable. This notion is supported by findings in one study of adolescents that found that lower inhibitory control, as rated by a self-report questionnaire, was associated with higher levels of pain catastrophizing [8]. Additionally, other studies have shown that poorer inhibitory control is associated with lower pain tolerance in other clinical samples, [9, 10] while better inhibitory skills are associated with lowered pain perception [9]. However, these studies were not conducted in the context of migraine.

The relationship between inhibitory control and pain catastrophizing may be particularly salient among individuals with migraine given that migraine is associated with frontal lobe abnormalities, a region implicated in inhibitory control [11, 12], including greater volume of white matter hyperintensities, reduced white matter structural integrity, and lower gray and white matter volume [13–18]. Moreover, recent work among individuals with migraine shows an association between higher levels of pain catastrophizing and brain abnormalities in areas associated with pain processing [19]; the areas involved overlap with those implicated in inhibitory control (i.e., prefrontal cortex; e.g., [11, 12]). Despite these possible links between inhibitory control and pain catastrophizing in migraine, no study has directly explored this potential interaction in relation to migraine pain intensity, duration, and frequency.

Therefore, the purpose of the current study is to examine whether inhibitory control interacts with pain catastrophizing to predict migraine pain intensity, attack frequency and duration in women with migraine and overweight/obesity after controlling for age, anxiety, depression, and body mass index (BMI). It is hypothesized that lower inhibitory control, measured objectively using a computerized Stroop task, will interact with higher pain catastrophizing, measured by the Pain Catastrophizing Scale [3]), to influence migraine pain intensity, attack frequency, and duration. If supported, this would have important treatment and research implications given that impaired inhibitory control can be improved through training [20–22]. This, in turn, could diminish the impact of pain catastrophizing, thus, potentially, reducing migraine severity.

## Methods

### Participants and procedures

The current study is an analysis of baseline data from the Women’s Health and Migraine (WHAM) trial (clinicaltrials.gov NCT01197196) [23]. Participants included 128 women aged 18 to 50 who were overweight or obese (BMI = 25.0-49.9 kg/m^2^) with a neurologist-confirmed migraine diagnosis based on the International Classification of Headache Disorders (3rd Edition beta criteria), [1] who sought behavioral weight loss treatment to reduce their migraine headaches. Participants were not currently pregnant or nursing and had not changed or initiated medications used to treat migraine attacks, depression, or contraception within two months prior to beginning the trial. Once informed consent was provided, participants completed a diagnostic evaluation by a board-certified neurologist to confirm their migraine diagnosis and to determine the onset of their migraines. Lastly, participants had their weight status and baseline preventative medication usage confirmed by study personnel, completed questionnaires, which assessed both psychological variables, including pain catastrophizing, and demographic characteristics, and were instructed on completing a 28-day headache diary. Once the 28-day diary was completed and verified by study staff, participants returned to the research center and completed the computerized version of the Stroop task. The study protocol was approved by the Rhode Island Hospital Institutional Review Board (Providence, RI, USA).

### Measures

#### Headache characteristics and clinical features

##### Daily headache monitoring

A web-based headache diary application, designed by the research team for use on smartphones [23], was used to record participants’ migraine headaches data prior to bedtime for 28 consecutive days. All ratings were automatically transmitted to the research center via the diary application and included occurrence (yes/no), maximum headache pain intensity (0 “no pain” to 10 “pain as bad as you can imagine”), and duration (hours). Research staff conducted daily checks to determine if headache data was complete (if not, participants were contacted to complete needed information). For the purposes of the present study, participant data were summarized as frequency (assessed as number of migraine days/mo.), average maximum pain intensity, and average duration of individual attacks. Participants also provided information about their abortive medication usage during the headache monitoring period.

#### Pain Catastrophizing

##### Pain Catastrophizing Scale (PCS)

The 13-item PCS was used to measure catastrophic thinking related to pain [3]. The PCS generates a total score and 3 subscale scores including rumination, magnification, and helplessness during the experience ofpain in previous month. PCS total scores range from 0 to 52, with higher scores indicating greater pain catastrophizing. The PCS has established reliability and construct validity and has previously been used to evaluate levels of pain catastrophizing in individuals with migraine [3, 5, 7, 24].

#### Inhibitory Control

##### Modified Stroop

The modified Stroop task assesses inhibitory control and was conducted using Eprime Stimulus Presentation Software (Psychology Software Tools, Pittsburgh, PA). The test is comprised of three conditions: Word, Color, and Color-Word. In the Word condition, participants responded to the meaning of a target word (i.e., ‘red,’ ‘green,’ or ‘blue’ displayed in black), as quickly as possible. In the Color condition, participants responded to the colors in which an array of X’s were displayed (i.e., XXXX displayed in red), as quickly as possible. The Color and Word conditions assess processing speed. In the Color-Word condition, participants responded to the colors in which an incongruent color word were displayed (i.e., ‘RED’ displayed in green), as quickly as possible. The Color-Word condition assesses inhibitory control as participants must suppress the response of reading the word [25, 26]. Participants completed three 45-s blocks of each condition, in a counterbalanced order. For each condition, all targets were displayed until the participant provided a response. The reaction time (in milliseconds) for correct responses in the Color-Word condition was used as the measure of inhibitory control.

#### Anthropometric characteristics

Height and weight were measured using a wall-mounted Harpenden stadiometer (Holtain, Ltd., Crosswell, Crymyh, Pembs, UK) and calibrated digital scale (Tanita BWB 800: Tanita Corporation of America, Inc, Arlington Heights, IL, USA). Body mass index (BMI) was calculated from these measures using the formula: BMI (kg/m^2^) = weight (kg)/(height [m])^2^.

#### Demographic characteristics

Age, marital status, race, ethnicity, and level of education were assessed via questionnaire.

#### Psychological characteristics

##### Depression

The 20-item Center for Epidemiologic Studies Depression Scale (CES-D) assessed frequency of depressive symptoms over the past week [27]. CES-D scores range from 0 to 60 with higher scores indicating more severe depressive symptoms. Clinically significant depressive symptoms are scores that are ≥ 16. The CES-D has high internal consistency, reliability, and both predictive and concurrent validity with respect to pain [27, 28].

##### Anxiety

The 7-item Generalized Anxiety Disorder Scale (GAD-7) assessed severity of anxiety symptoms over the previous 2-week period [29]. Higher scores are indicative of greater levels of anxiety symptoms, with scores of 5, 10, and 15 serving as cutoff points for mild, moderate and severe anxiety symptoms, respectively. This measure is shown to accurately and reliably assess anxiety in primary care settings and the general population [29, 30].

### Statistical analyses

Descriptive statistics were generated to characterize the sample. Bivariate correlations, independent samples t-tests, and one-way analyses of covariance (ANOVA) were conducted to examine associations between demographic variables and inhibitory control to determine covariates for inclusion in the primary analysis. As a result, age was entered as a covariate in subsequent analyses. It is important to note that initial onset of migraine and use of preventative or abortive medications during the headache monitoring period were not associated with pain catastrophizing (total or subscale scores) or inhibitory control, and were therefore not included as covariates. Additional covariates included, depression and anxiety, to enable clearer examination of the potential influence of pain catastrophizing, independently or in conjunction with inhibitory control, and BMI, due its known association with pain catastrophizing [5, 31] and inhibitory control [32, 33]. Separate linear regression models were conducted to examine the independent and joint associations of pain catastrophizing (total score and subscales) and inhibitory control (Stroop Color Word RT) with each of the migraine severity components (i.e. pain intensity, attack frequency and duration). All tests of statistical significance were two-tailed and tolerance for Type 1 error was set at α = 0.05. This study involved a secondary analysis of baseline data collected as part of an ongoing randomized controlled trial.

## Results

### Characteristics of the sample

Twenty-one individuals were excluded from analyses due to their poor performance level (i.e., <50% correct responses on the Stroop Color-Word test); participants were excluded as poor performance may have been due to reduced effort or understanding of the task and the cut-off used is similar to previous studies using similar cognitive tasks [34–36]. An additional 2 participants were excluded for not completing the 28-day headache protocol, resulting in a total sample of 105 participants. Table 1 shows demographic, anthropometric, and pain catastrophizing scores of the sample. On average, participants were 38 years of age with severe obesity (M_BMI_ = 35.1 ± 6.5 kg/m^2^). Approximately one-fifth (20.8%) of participants were non-White and 16.0% were Hispanic. The majority of participants had a college degree (40.6%) or had completed some college (30.2%) while 18.9% completed additional graduate/professional training; 10.2% of participants had a high school degree or less than 12 years of education. On average, participants reported having a migraine attack on 8 days over the 28-day monitoring period and rated the maximum pain intensity per attack on average as moderate or 6 out of 10. With respect to initial migraine onset, 39.3% reported during childhood, 26.2% during teenage years, 31.8% during their 20’s to 30’s, and 2.8% over age 40. None of the participants reported having a migraine attack on the day of cognitive testing. Participants averaged 93.8 ± 01% accuracy and 1203.5 ± 275.8 ms reaction time on the Color-Word condition of the Stroop test. Of the 105 included participants, 21.5% were using preventative medications at baseline. Additionally, the vast majority of participants used abortive medications (90.7%) during the headache monitoring period including non-steroidal anti-inflammatory drugs (NSAIDs; 66.4%), Excedrin (24.3%), Triptan (33.6%), and other medications (36.4%) which includes opiods (14%), Butalbital (15.9%), and herbal medications, anti-convulsants, muscle relaxants, analgesics, and anti-depressants (.9% each). Independent samples t-tests revealed no significant differences in inhibitory control or pain catastrophizing (total score and subscales) between individuals with and without aura (p’s > .31); therefore, these groups were analyzed together.

**Table 1:** Characteristics of the sample

Demographic and anthropometric characteristics	
Age, years, mean (SD)	38.0 (1.2)
Race (% of participants)	
African American	9.3%
White	78.5%
Other	12.2%
Ethnicity (% of participants)	
Non-Hispanic	84.0%
Hispanic	16.0%
Education (% of participants)	
High school or less than 12 years	10.2%
Some College	30.2%
College Degree	40.6%
Graduate/Professional Education	18.9%
Body mass index, kg/m^2^, mean (SD)	35.1 (6.5)
Migraine Initial Onset (% of participants)	
Childhood	39.3%
Teenage years	26.2%
Early Adulthood (20’s – 30’s)	31.8%
Over age 40	2.8%
Migraine Severity	
Migraine Parameters	
Frequency, mean (SD) days/mo. (# episodes)	5.5 (2.6)
Migraine Days, mean (SD)	8.4 (4.4)
Intensity, mean (SD) on scale of 1 to 10	5.9 (1.4)
Duration, mean (SD) hours	20.0 (14.2)
Medication Use (% of participants using)	
Migraine Preventative Medications	21.5%
Abortive medications	90.7%
NSAID	66.4%
Excedrin	24.3%
Triptan	33.6%
Other	36.4%
Pain Catastrophizing (mean, SD)	
Total Score	22.6 (11.0)
Rumination	9.1 (4.3)
Magnification	4.3 (3.1)
Helplessness	9.2 (4.9)
Inhibitory Control	
Stroop Color-Word Reaction Time, mean (SD) msec	1203.5 (275.8)
Stroop Color-Word (% correct responses)	93.8 (0.1)

### Independent and joint associations of pain catastrophizing and inhibitory control with migraine severity components

Regression models examined independent and joint associations of pain catastrophizing and inhibitory control with 3 indices of migraine severity (headache pain intensity and attack frequency and duration) controlling for age, depression, anxiety, and BMI.

#### Migraine Pain Intensity (Table 2 Column 1)

**Table 2:** Linear regressions examining pain catastrophizing, inhibitory control, and their interactions in relation to migraine severity as measured by attack frequency, pain intensity and duration

	Pain Intensity					Duration					Attack Frequency				
	B	*SE*	*β*	*p*	*R* ^*2*^	B	*β*	*SE*	*p*	*R* ^*2*^	B	*β*	*SE*	*p*	*R* ^*2*^
PCS Total					.12					.11					.13
Age	.0003	.02	.002	.99		-.15	.18	-.09	.40		-.06	.05	-.12	.26	
BMI	-.03	.02	-.13	.22		-.29	.23	-.13	.20		-.12	.07	-.17	.09	
Depression	.01	.04	.04	.73		.15	.41	.05	.71		.15	.13	.15	.23	
Anxiety	.05	.04	.15	.22		.20	.37	.06	.60		.16	.11	.17	.16	
PCS-Total	-.10	.06	-.78	.08		.05	.57	.04	.93		-.24	.18	-.60	.17	
IC	-.002	.001	-.33	.10		-.001	.01	-.03	.90		-.003	.003	-.17	.40	
PCS-Total*IC	.0001	.00005	1.02	.04		.0002	.0005	.23	.64		.0002	.0001	.63	.19	
Rumination					.11					.09					.16
Age	-.001	.02	-.008	.94		-.17	.18	-.10	.34		-.06	.05	-.11	.26	
BMI	-.03	.02	-.11	.28		-.30	.23	-.14	.19		-.11	.07	-.16	.12	
Depression	.02	.04	.05	.68		.20	.42	.06	.64		.15	.12	.14	.23	
Anxiety	.05	.04	.15	.22		.22	.38	.07	.55		.17	.11	.18	.12	
Rumination	-.30	.16	-.91	.06		-.89	1.58	-.27	.58		−1.00	.47	-.97	.18	
IC	-.002	.001	-.34	.11		-.005	.01	-.10	.64		-.004	.003	-.28	.18	
Rumination*IC	.0003	.0001	1.05	.04		.001	.001	.48	.36		.001	.0004	.88	.08	
Magnification				.12					.14					.13	
Age	-.001	.02	-.004	.97		-.09	.18	-.05	.61		-.06	.06	-.11	.28	
BMI	-.02	.02	-.11	.29		-.29	.22	-.13	.19		-.12	.07	-.18	.08	
Depression	.02	.04	.05	.70		.04	.41	.01	.92		.15	.13	.14	.24	
Anxiety	.05	.04	.16	.17		.19	.36	.06	.61		.16	.11	.16	.17	
Magnification	-.30	.20	-.64	.15		-.43	2.02	-.09	.83		-.78	.63	-.53	.23	
IC	-.001	.001	-.18	.22		-.004	.008	-.08	.58		-.002	.002	-.10	.52	
Magnification*IC	.0003	.0002	.86	.07		.002	.002	.44	.34		.001	.0005	.61	.19	
Helplessness					.12					.09					.11
Age	-.002	.02	-.01	.92		-.21	.18	-.12	.26		-.07	.06	-.12	.24	
BMI	-.03	.02	-.12	.26		-.22	.23	-.10	.33		-.12	.07	-.17	.10	
Depression	.02	.04	.05	.65		.24	.41	.07	.56		.16	.13	.15	.22	
Anxiety	.05	.04	.15	.20		.27	.37	.09	.46		.15	.11	.15	.20	
Helplessness	-.18	.13	-.63	.16		.97	1.30	.33	.46		-.13	.40	-.15	.74	
IC	-.001	.001	-.26	.18		.006	.01	.11	.57		-.0003	.003	-.02	.92	
Helplessness*IC	.0002	.0001	.86	.08		-.0003	.001	-.16	.75		.0001	.0003	.21	.66	

*BMI* Body mass index, *PCS* Pain Catastrophizing Scale, *IC* Inhibitory Control

There were no independent associations between inhibitory control or pain catastrophizing and pain intensity (*p*’s > .39). However, inhibitory control interacted with pain catastrophizing total (β = 1.02, SE = .00005, *p* = .04) and rumination (β = 1.05, SE = .0001, *p* = .04) subscale scores to predict headache pain intensity, such that the association between pain catastrophizing and pain intensity became more positive at lower levels of inhibitory control.

#### Migraine Attack Duration (Table 2, Column 2)

Higher pain catastrophizing total (β = .241, SE = .14, *p* = .03) and magnification subscale (β = .311, SE = .51, *p* < .01) scores were significant independent predictors of longer individual attack duration. Measures of inhibitory control and the interaction of pain catastrophizing with inhibitory control did not influence average duration of individual migraine attacks (*p*’s > .34).

#### Migraine Attack Frequency

There were no independent or joint associations for inhibitory control and pain catastrophizing in relation to headache attack frequency (*p*’s > .08).

## Discussion

This study examined whether pain catastrophizing interacted with objective measures of inhibitory control to predict migraine severity components among women with migraine and overweight/obesity. The primary finding is that pain catastrophizing [overall and specific sub-components (i.e. rumination and helplessness)] interacted with inhibitory control to predict headache pain intensity, such that increased catastrophizing was associated with higher levels of pain intensity when inhibitory control was reduced. In contrast, no interaction between pain catastrophizing and inhibitory control was found in relation to headache frequency or duration of individual headache attacks.

These findings build on previous research showing that higher levels of pain catastrophizing are associated with migraine attacks that are more severe and disabling [5–7, 37] by highlighting the potential contribution of poorer inhibitory control. This contribution seems to be particularly important in the context of degree of pain intensity within an acute attack. This finding coupled with the lack of association with attack frequency or duration suggests that lower inhibitory control exacerbates the adverse effect of pain catastrophizing after the initiation of an attack, but does not necessarily increase likelihood of attack occurrence or impact pain duration. Alternatively, it is possible that the interaction of IC and PC might be stronger in relation to the more subjective (i.e. degree of perceived pain) versus objective (i.e. occurrence and duration of pain) components of migraine severity.

The present study advances previous work focused on catastrophizing and/or inhibitory control in the context of pain [5–10] by demonstrating the interaction of these variables in relation to frequency, duration, and intensity of a specific pain condition (migraine). Further, our examination of the relationship between inhibitory control and specific components of pain catastrophizing (e.g., rumination) provides greater insight into the inhibitory control-pain catastrophizing link. Specifically, rumination in the context of migraine refers to difficulty inhibiting pain-related thoughts, and it would follow that reduced inhibitory control might exacerbate this difficulty.

This study also has important clinical implications as research has shown that inhibitory control may be improved through computerized cognitive training [20–22], which might help attenuate any adverse effects of pain catastrophizing on pain intensity. Future studies should examine whether improvement in inhibitory control through training could lead to less severe migraine attacks via reduced pain catastrophizing. This study has several methodological strengths including the large sample size and the use of smartphone diaries for near real time monitoring of daily headache activity over a 4-week period. Additionally, the use of computerized measures of inhibitory control reduces the possibility of measurement error and bias.

This study must also be viewed in light of certain limitations and considerations for future research. Given the cross-sectional study design, we cannot infer causality in the observed associations. Thus, future research involving prospective designs that can provide insight regarding causality and underlying mechanisms are needed. Although this sample is advantageous in that higher levels of pain catastrophizing and lower inhibitory control may be expected among overweight/obese individuals [5, 31–33], future studies should examine whether these results generalize to more diverse samples in terms of weight status as well as gender, given that this sample was comprised solely of female participants. Another limitation is that the PCS items are not specific to migraine and thus may have captured other types of pain in addition to migraine. The presence of other pain-related conditions was not assessed in this study, which could have affected the results. Additionally, other factors that may contribute to pain catastrophizing and inhibitory control in this sample, such as sleep quality and duration, were not examined; future studies should examine the observed relationships in the context of other such factors.

## Conclusions

In conclusion, the present study showed that lower inhibitory control interacted with higher levels of pain catastrophizing to predict greater pain intensity during migraine headache attacks. Future prospective studies are needed to determine whether improvement in inhibitory control through training could lead to less severe attacks via attenuation of pain catastrophizing.
